# Disentangling trust of patients with rare cancer in their healthcare professionals and the healthcare system: a qualitative interview study

**DOI:** 10.1007/s11764-023-01531-w

**Published:** 2024-01-16

**Authors:** Barbara C. R. Simons, Marij A. Hillen, Johanna W. M. Aarts, Jacqueline M. Tromp, Eline de Heus, Saskia F. A. Duijts

**Affiliations:** 1 Department of Research and Development, Netherlands Comprehensive Cancer Organisation (Integraal Kankercentrum Nederland, IKNL), Rijnkade 5, 3511 LC Utrecht, The Netherlands; 2 Department of Medical Psychology, Amsterdam University Medical Centers, University of Amsterdam, Meibergdreef 9, 1105 AZ Amsterdam, The Netherlands; 3 Department of Obstetrics and Gynaecology, Amsterdam University Medical Centers, University of Amsterdam, Meibergdreef 9, 1105 AZ Amsterdam, The Netherlands; 4 Gynaecological Oncology, Cancer Center Amsterdam, De Boelelaan 1118, 1081 HV Amsterdam, The Netherlands; 5 Department of Medical Oncology, Amsterdam University Medical Centers, Meibergdreef 9, 1105 AZ Amsterdam, The Netherlands; 6 Department of Medical Oncology, Radboud University Medical Center, Geert Grooteplein Zuid 10, 6525 GA Nijmegen, The Netherlands; 7 Cancer Treatment and Quality of Life, Cancer Center Amsterdam, De Boelelaan 1118, 1081 HV Amsterdam, The Netherlands

**Keywords:** Rare cancer, Trust, Supportive care, Oncology, Patient-physician relationship

## Abstract

**Purpose:**

Patients with a rare cancer face challenges, e.g., delayed diagnosis, that may affect trust in the healthcare system and the healthcare professionals (HCPs) involved. This study aimed to explore trust of patients with a rare cancer in their HCPs and the healthcare system.

**Methods:**

Semi-structured interviews were conducted with 20 purposively sampled patients with a rare cancer. The interview guide included topics related to trust, including level, development, barriers and facilitators, importance, and trust dimensions. Thematic analysis was conducted with use of Atlas.ti.

**Results:**

The mean age of patients was 50 years, 60% were female, and 70% were highly educated. Three themes were constructed: (1) “*Confirmed* expertise is a prerequisite of trust.” Patients need *confirmation* of their HCPs’ expertise, as it could not be assumed due to the rarity of their cancer; (2) “Trust depends on the adequacy of information and *how* it is provided.” Limited information about rare cancer reduced patients’ trust in health care, whereas interpersonal trust was mainly affected by *how* HCPs provided information; and (3) “Trust is built on properly coordinated and supportive care.” Proper organization and cooperation within and between hospitals, and integration of supportive care, enhanced trust.

**Conclusion:**

Patients with a rare cancer experience challenges that influence trust in HCPs and the healthcare system. Further research should examine trust among subgroups of patients with a rare cancer, to enable development of tailored interventions.

**Implications for Cancer Survivors:**

HCPs may improve trust by focusing on expertise, effective information provision, proper coordination of care, and provision of adequate supportive care.

**Supplementary Information:**

The online version contains supplementary material available at 10.1007/s11764-023-01531-w.

## Introduction

A cancer type is defined as “rare” if there is an incidence of < 6/100.000 people per year [1]. In total, 24% of the total number of new cancer diagnoses constitute rare cancers [1, 2]. Examples of rare cancer types include head and neck cancers, sarcomas, and certain cancers of the urinary or genital tracts. Cancers among children and common types of cancer at an unusual location in the body (e.g., a melanoma in the eye) can also be considered rare. Rare cancers present unique challenges to patients, healthcare professionals (HCPs), and the healthcare system, such as the difficulty to conduct clinical studies due to the limited number of patients [1]. Moreover, knowledge, information, expertise, and evidence are not always widely available [3, 4]. This can lead to misdiagnosis, delayed diagnosis, the absence of clear treatment guidelines, and uncertainty regarding treatment outcomes [1, 4]. Patients with a rare cancer face several disadvantages and challenges. As a group, they have lower survival rate and experience lower quality of life, compared to patients with a more common cancer type [5, 6]. They may also experience higher unmet needs regarding information provision and finding their way through the healthcare system [7, 8]. These issues might lead to psychosocial concerns and could affect patients’ trust in their medical specialists, other HCPs, and the healthcare system [7].

Trust is an essential component of any patient-provider relationship, especially when facing a complex and uncertain diagnosis, such as cancer [9, 10]. Stronger trust is associated with improved treatment adherence, patient satisfaction, and self-reported health outcomes [11, 12]. Trust may also facilitate communication between the patient and the HCP and reduce the probability of patients requesting a second opinion [11]. Trust can be divided into interpersonal trust and public trust. Patients’ interpersonal trust in the HCPs involved in their patient trajectory can be defined as “the optimistic acceptance of a vulnerable situation, in which the patient believes the provider cares for his or her best interests” [13]. Previous research from Hillen et al. [14] distinguished several dimensions of interpersonal trust, i.e., fidelity (meaning that the patient is confident that the professional acts in their best interest), competence, honesty, and caring. Patients’ trust in the healthcare system (“public trust”) can be defined as “being confident that you will be adequately treated when you are in need of health care” [15]. Six dimensions of public trust were previously distinguished among patients in general: HCP’s focus on the patient, HCP’s expertise, information supply and communication, quality of care, quality of cooperation between HCPs, and macro-level policies. It has been suggested that trust in the HCPs’ expertise and experience, and in the healthcare system, may be of particular importance among patients with a rare cancer [16].

Previous studies on trust and related dimensions have primarily focused on patients with cancer in general. However, considering the specific disadvantages and challenges patients with a rare cancer may experience, trust might develop and be affected differently in this group. Therefore, the aim of this study was to explore trust of patients with a rare cancer in their HCPs and the healthcare system.

## Methods

### Study design

We applied a qualitative interpretive design with an inductive approach. Semi-structured interviews were conducted in April–May 2023. The COnsolidated criteria for REporting Qualitative research (COREQ) checklist was used to ensure explicit and comprehensive reporting [17] (Supplementary file 1). The Medical Ethics Committee (METC) of Amsterdam University Medical Centers (AUMC) and the Institutional Review Board (IRB) of the Netherlands Cancer Institute (NCI) reviewed the study and confirmed that the Medical Research with Human Subjects Act (WMO) did not apply. Therefore, official approval of the study was not required (reference: METC2022.0893; IRBd23-087).

### Study sample

Patients were eligible for participation if they were diagnosed with a rare cancer, were 18 years or older, and were able to speak and understand Dutch. Based on the information provided by the patient and/or medical specialist, the researcher (BS) verified eligibility according to the RARECARENet list [18]. To ensure variation and create an all-encompassing view, purposive sampling was used [19]. As this was a first exploration within this patient group, variation was sought in rare cancer type and in sociodemographic factors (i.e., age, gender, and education level), as these factors may be associated with trust in the HCPs and healthcare system [11, 20].

### Study procedures

We recruited patients through medical specialists of AUMC and the NCI, relevant (patient) organizations or platforms (Dutch Federation of Cancer Patients Organisations (NFK) and Dutch Rare Cancer Platform (DRCP)), and via social media (i.e., Facebook, Twitter, and LinkedIn). Within the participating hospitals, medical specialists informed patients about the study. If a patient was interested, the medical specialist provided the patient’s contact details to the researcher (BS). Patients who were informed about the study through other sources (e.g., NFK, DRCP, social media) could contact the researcher directly via email. In case a patient expressed interest, the researcher contacted the patient by phone to provide more information. An information package was then sent by mail. The information package included an information letter, a short questionnaire with socio-demographic and disease-related questions, and an informed consent form. Upon receiving informed consent, the researcher scheduled an interview with the patient, which took place at the patient’s home or through video conferencing, based on the patient’s preference. Recruitment of patients continued until the researchers perceived data saturation was reached, i.e., when no new codes or themes emerged from the data [21].

### Data generation

An interview guide was developed to ensure that all relevant topics were addressed (see supplementary file 2). The interview guide was based on literature and further developed with input from researchers in the fields of trust and rare cancer. It included questions regarding the following: (1) the patient’s disease trajectory, (2) trust in general, (3) the level and development of trust (i.e., patients’ degree of trust in the medical specialist, in other HCPs, and in the healthcare system, as well as whether and how trust developed during the trajectory), (4) barriers and facilitators of trust, (5) the importance of trust, and (6) relevant dimensions of “interpersonal” and “public” trust. Before data generation started, the researcher (BS) conducted two pilot interviews to become familiarized with the interview guide and to test its applicability [22]. Afterwards, minor adjustments were made to the wording of questions. Interviews were conducted by a member of the research team (BS, graduate student, health sciences, trained in qualitative research methods). A second member of the team was present during the interviews to operate the recording equipment and reflect on the interviews afterwards. Interviews were audio recorded, transcribed verbatim, and anonymized before data analysis.

### Data analysis

We performed data analysis in April–June 2023, largely in parallel with data generation. Thematic analysis was performed on interview data, according to the 6-stage framework developed by Braun and Clarke [23]. This allowed us to openly identify and interpret patterns in patients’ experiences regarding trust in their HCPs and the healthcare system [24]. Two members of the research team (BS, EZ) were involved in the coding process to ensure researcher triangulation [25]. The first three transcripts were individually coded by these two members, after which they discussed these codings. After the development of the initial code tree, this process continued by double coding and discussing every fifth transcript. During the coding process, the constant comparative method was used [26]. To increase confirmability, the research process and findings were discussed each week with senior members of the research team (SD, MH) during data generation and analysis [27]. After data analysis, a meeting was arranged to reach an agreement on the interpretation of interview data and the constructed themes were discussed with project members (BS, MH, EdH, SD). Atlas.ti 23.1.1 was used to support data analysis [28]. An overview of the codes can be found in supplementary file 3. Descriptive statistics were calculated to describe the participant group.

## Results

Of the 32 interested patients, 27 returned the completed documents from the information package. One patient reported not being able to proceed because of deteriorating health; four patients could not be reached. Of the 27 patients who returned the documents, patients were purposively selected and interviewed until data saturation was observed (*N* = 20). Informed consent was obtained from all individual participants included in the study. The mean age of patients was 50 years (range 30–83), 60% were female, and the majority were highly educated (70%), see Table 1. Interviews lasted on average 57 min (range 42–77).

**Table 1:** Patient characteristics

	*n* (%)
*Gender*	
Male	8 (40%)
Female	12 (60%)
*Age*	
30–39	3 (15%)
40–49	3 (15%)
50–59	4 (20%)
60–69	4 (20%)
70 +	6 (30%)
*Education level*^a^	
Low	2 (10%)
Medium	4 (20%)
High	14 (70%)
*Cancer type*	
Cancer of the endocrine organs	1 (5%)
Central nervous system cancer	1 (5%)
Digestive cancer	2 (10%)
Female genital cancer	3 (15%)
Head and neck cancer	3 (15%)
Hematological cancer	1 (5%)
Male genital cancer	2 (10%)
Neuroendocrine carcinoma	1 (5%)
Sarcoma	5 (25%)
Skin cancer	1 (5%)
*Years since diagnosis*	
< 1	6 (30%)
*1– < 3*	10 (50%)
3– < 5	2 (10%)
> 5	2 (10%)
*Hospital of diagnosis*	
Regional hospital	7 (35%)
Academic hospital or CoE^b^	13 (65%)
*Hospital of treatment*	
Regional hospital	3 (15%)
Academic hospital or CoE^b^	17 (85%)
*Currently receiving treatment*	
Yes	6 (30%)
No	14 (70%)

^a^Low—all years of primary school, first 3 years of senior general secondary education and pre-university secondary education, all prevocational secondary education including lower secondary vocational training and assistant’s training

Medium—upper secondary education, (basis) vocational training, middle management and specialist education

High—associate degree programs, higher education (bachelor/master programs), doctoral degree programs

^b^*CoE* Centre of Expertise

### Constructed themes

Three themes were constructed from the data: (1) *Confirmed* expertise is a prerequisite of trust; (2) Trust depends on the adequacy of information and *how* it is provided; and (3) Trust is built on properly coordinated and supportive care.

#### Theme 1: confirmed expertise is a prerequisite of trust

For the majority of patients, expertise was an important component of trust, as well as one of the most frequently mentioned reasons for trusting their HCP. Patients considered characteristics related to expertise, such as the HCP’s knowledge, experience, interpersonal skills, and confidence, to be beneficial for trust. Patients considered it important to know whether HCPs have expertise but also valued HCPs’ honesty about any lack of knowledge or skills regarding their specific rare cancer type.

*I think expertise is of course essential [for trust]. And honesty regarding one’s own expertise. You see, when it comes to rare cancers, one should be able to say ‘well, I just don’t know’.*

– Male, 76 years old, hematological cancer

Some patients indicated that HCPs’ expertise was difficult to verify, due to limited available information or because of their own lack of medical knowledge. In these situations, receiving *confirmation* of the HCPs’ expertise specifically increased patients’ trust. Patients derived expertise for example from the HCP’s title (e.g., renowned specialist or professor), recommendation or confirmation from other HCPs or patients, reading information about a medical specialist, or receiving treatment at an appointed Centre of Expertise (CoE).

*My friend said: ‘Well, if you need an oncologist or gynaecologist, you definitely got the very best’. I thought: ‘In that case, it’s okay.’ I don’t know anything about the medical realm, it’s not my profession. My friend tells me these two [medical specialists] are very good. So, I completely entrusted myself to that.*

– Female, 40 years old, neuroendocrine carcinoma

A few patients experienced situations where professionals’ lack of knowledge and expertise negatively affected their trust. These situations included failing to recognize symptoms specific for their rare cancer or refusing a request to (re-)examine a symptom through (physical) examination. Some patients mentioned that a HCP could not be blamed if symptoms were not immediately recognized, because their tumor type was so rare. However, they emphasized that HCPs can enhance trust by taking complaints seriously and taking action if required, for example, by conducting an examination or referring to a more specialized hospital (i.e., a CoE).

*It is basically [the HCP’s] lack of knowledge, or – in my case – not considering rare conditions. Which means you can’t add up everything and think, ‘there might be something else going on here. Let’s refer this woman to an academic hospital.’*

– Female, 33 years old, genital cancer

Almost all interviewees in this study were referred to a CoE or an academic hospital, at diagnosis or immediately after. Overall, their trust in HCPs in these centers was high.

*Maybe it makes a difference that [hospital] is a university hospital. That may subconsciously adds extra trust [in HCPs].*

– Male, 55 years old, head and neck cancer

At a macro level, interviewees indicated that their trust would be strengthened if further development of expertise regarding rare cancers could be realized. Patients additionally stated that a lack of (clear) guidelines or information on their treatment negatively affected their trust. Yet, trust was enhanced if in such situations, HCPs collaborated and/or asked for advice from experts (nationally or internationally) to confirm their approach. Some patients mentioned that trust in the healthcare system would improve if more research was performed and if resources (e.g., money and support) were obtained to (further) develop the organization of care for patients with rare cancer.

*Not many people have this type of cancer (…). Therefore, they also consulted the cancer centre in [place abroad], for their recommendations regarding treatment methods. (…) That definitely increased my trust.‬‬‬‬‬‬‬‬‬‬‬‬‬‬‬‬‬‬‬‬‬‬‬‬*

– Female, 40 years old, neuroendocrine carcinoma

#### Theme 2: trust depends on the adequacy of information and how it is provided

A majority of interviewees indicated that honesty was one of the most important dimensions of trust in their HCP. They perceived honesty specifically if HCPs were transparent about available information (e.g., general information about their rare cancer type or treatment options), even if that information was uncertain.

*When they [oncologists] say: ‘Read this article online. It contains results regarding the chemotherapy. But be aware that it’s not a high quality study, because the sample size is too small’. But they still tell you that, because that’s what you want. You are really seeking some guidance.*

– Female, 44 years old, cancer of the endocrine organs

Importantly, interpersonal trust was mainly affected by *how* information was provided. Regardless of whether information is being available or not, the way HCPs communicated the information to them determined patients’ trust. Almost every interviewee mentioned that their trust was strengthened if a HCP had good communication skills, conveying calmth, transparency, directness, and conviction.

*What I believe really boosted my trust (…), was when the head of the department calmly explained my diagnosis to me (…) She ensured clarity for me as the patient and my relatives about what was going on. I think that is when I realized ‘now it is going to be okay, now we can let go’.*

– Female, 33 years old, genital cancer

Most interviewees experienced the information available (e.g., online, provided by HCPs or folders) to them as insufficient. This concerned general information about their tumor type, adequate treatment, their prognosis, specific rare cancer guidelines, or where to go to receive optimal care (i.e., CoE for their tumor type). This lack of information affected their trust in the healthcare system and sometimes caused insecurity or a perceived lack of control over the situation.

*(…), because I have a very rare tumor. So they [medical specialists] don’t have a lot of experience with it. They said: ‘We don’t know much about it, so we can’t say much about it’. But I still need a course of action. And then you kinda have the idea that you have to figure that out on your own.*

– Female, 48 years old, central nervous system cancer

A lack of adequate information could also affect patients’ interpersonal trust. Patients indicated that their trust decreased if they perceived having incorrect or incomplete information. As a result, patients would be reluctant to consult their HCP or be skeptical and controlling towards the given information.

*So, I did not have a lot of trust in that physician, simply because I had my doubts about certain things [after receiving wrong information]. So I didn’t like going there.*

– Female, 44 years old, cancer of the endocrine organs

As a lot is still unknown regarding rare cancers, some patients indicated having searched for (international) studies to gather information and sharing the information with their HCPs. They stated that it contributed to their trust if HCPs were receptive towards such information and willing to discuss the information with them.

*I did a lot of research and consulted studies, including ones from abroad. I shared the conclusions with the doctors, and they took me very seriously in that regard. It wasn’t like ‘oh, here is another difficult patient who thinks he knows better’. They engaged in a genuine conversation with me and wanted to hear my perspectives (…). That’s when trust really grows.*

– Male, 50 years old, sarcoma

#### Theme 3: trust is built on properly coordinated and supportive care

Many interviewees mentioned that the hospital’s organization of care affected their trust in the healthcare system. Patients’ trust increased throughout the disease trajectory if care was coordinated properly. Perceived indicators of proper coordination were, for example, short waiting times, adequate collaboration and knowledge sharing between HCPs involved, and efficient scheduling of multiple hospital appointments.

*And, they are a team that collaborates. They get perspectives from different specialties and everyone knows about my case. I don’t have to explain the story over and over again. It all comes across as very organized and professional. That also gives me trust. ‬‬‬‬‬‬‬‬‬‬‬‬‬‬‬‬‬‬‬‬‬‬‬‬‬‬‬‬‬‬‬‬‬‬‬‬‬‬‬‬‬‬‬‬‬‬‬‬‬‬‬‬‬‬‬‬*

– Female, 52 years old, sarcoma

Moreover, patients indicated that a continuous treatment relationship with the same HCP in the hospital was important in building trust.

*Conducive [to trust] was that I saw the same doctor all the time. When I look at my peers (…), they see different doctors all the time. So they have to explain everything all over again because it’s a very rare cancer type. These doctors just know very little about it. And because I talk to these people, I think to myself: ‘The hospital where I am treated is very good in this regard’. (…) So yes, seeing the same doctor who knows your file and prepares himself… Yes, that strengthens trust.*

– Female, 71 years old‬‬‬‬‬‬‬‬‬‬‬‬‬‬‬‬‬‬‬‬‬‬‬‬‬‬‬‬‬‬‬‬‬‬‬‬‬‬‬‬‬‬‬‬‬‬‬‬‬‬‬‬‬‬‬‬‬‬‬‬‬‬‬‬‬‬‬‬, skin cancer‬‬‬‬‬‬‬‬

Many patients had to deal with multiple hospitals, due to referral from regional to academic hospitals for diagnostic procedures or due to treatment in various hospitals. Trust increased if the referral between hospitals took place efficiently. Patients’ trust was impaired when there was insufficient collaboration *between* hospitals or if medical records were transferred inefficiently or incorrectly. Respondents indicated in addition that trust was reduced if collaboration between HCPs *within* the hospital fell short.

*I had to correct errors [throughout the process] a lot. The appointments were incorrect, the biopsy had the wrong name and medical data were not transferred. This gave me quite a lot of stress, because I couldn’t trust it [the process].‬‬‬‬‬‬‬‬‬‬‬‬‬‬‬‬‬‬‬‬‬‬‬‬‬*

– Male, 55 years old‬, sarcoma

The challenges patients experienced during their rare cancer trajectory (e.g., having to arrange a lot themselves, being assertive) created a need for (interpersonal) support. For many patients, trust was enhanced if a HCP had caring qualities such as showing empathy, listening, making a connection, and providing a safe environment. Further, HCPs being committed, approachable, not treating the patient as a number, and putting the patient’s best interests first were additional aspects that were mentioned in the context of receiving support and increased trust.

*[For trust] you need to feel good about someone. And [have] a good patient-physician relationship. You need to feel like you’re not just a number. And, ehm, in this respect, I am extremely well situated with my oncologist.‬‬‬‬‬‬‬‬‬‬‬‬‬‬‬‬‬‬‬‬‬‬‬‬‬‬‬‬‬‬‬‬‬‬‬‬‬‬‬‬*

– Male, 55 years old, head and neck cancer.

On the contrary, some patients indicated that caring behavior was not essential for having trust in their medical specialist, as they considered medical expertise most important.

*But I don’t really need the doctor to be very caring. He has to do his job right [to receive trust].*

– Female, 63 years old, sarcoma

Some patients received support from a fixed point of contact during their trajectory. Such a point of contact could be a nurse navigator, nurse practitioner, general practitioner, or, in some cases, the medical specialist him/herself. Patients’ trust in the professional who was fulfilling this role was generally high. Interviewees indicated that such a person provided guidance, answered questions, provided further explanation, and took action throughout their trajectory if needed. However, not every patient had a fixed point of contact. Patients who were lacking this specific person indicated to have missed this form of support. In contrast, patients for whom a point of contact was part of the provided care indicated that this increased their trust in the healthcare system.

*Yes, I think a fixed point of contact does that [increase trust], to be honest. It is nice to have one person who knows everything about you. And who you can always rely on (…). Because you feel like a number throughout the whole process, everyone is busy. Then it’s great to have one person, who at least gives the impression that you are the only patient.*

– Male, 55 years, head and neck cancer

Some patients mentioned that their trust in the healthcare system might increase through further development of (supportive) care for patients with a rare cancer, for example, through tailoring to patients’ needs or development of guidelines.

*To some extent, I trust the system. But I know that when it comes to rare cancers, there is not enough awareness in the healthcare system to properly adapt it to the needs of the patients. There are collaborative networks, but I don’t think they function optimally yet (…).If that had functioned better, it would have improved my trust.*

– Male, 76 years old, hematological cancer

## Discussion

### Main findings

The aim of this study was to explore trust of patients with a rare cancer in their HCPs and the healthcare system. Patients highlighted the importance of expertise for trust in their HCPs, yet found such expertise difficult to assess. Receiving explicit *confirmation* of the HCP’s expertise (e.g., from a recommendation) enhanced patients’ trust. Further, a perceived lack of (adequate) information reduced patients’ trust in healthcare, whereas interpersonal trust was mainly affected by *how* the information was provided. Finally, patients’ trust in the healthcare system was strengthened by having a fixed point of contact which provided support and through efficient organization and cooperation within and between hospitals and HCPs.

### Interpretation of the findings

Expertise appeared to be an important dimension of trust among patients with a rare cancer. This is in line with previous findings in which expertise was identified as a dimension of interpersonal as well as public trust [14, 29]. However, in these studies, it was reported that patients often presuppose that a HCP’s medical skills are present, whereas in the current study, patients more explicitly mentioned expertise (regarding their rare cancer type) as an important requirement for having trust in their HCPs [30]. The emphasis patients placed on expertise may be explained by the lack of available medical knowledge regarding specific rare cancer types [3, 4], making it less self-evident that HCPs have the required expertise. In line with previous findings, it was found that patients’ trust increased if the HCP’s expertise was confirmed [31]. This might be explained by findings from Roberts et al. [32], who stated that patients can be limited in their ability to assess physicians’ technical skills and therefore tend to base their assessment of competence on interpersonal skills (i.e., communication and interaction) [32]. However, given the less self-evident nature of expertise for patients with a rare cancer, technical skills are crucial. This could mean that interpersonal skills on their own are insufficient to enable patients to assess the HCP’s expertise. As a result, patients may need more explicit indicators of (technical) expertise, such as confirmation, to gain trust.

Patients’ interpersonal trust was mainly affected by *how* a HCP provided information, and patients valued the HCPs’ honesty about limitations and uncertainties of their knowledge. This is consistent with previous research where “communication and information provision” was found to be a dimension within public trust and “honesty” a dimension within interpersonal trust [14, 29]. However, previous research [33] showed that physicians may be reluctant to share limited and uncertain information, fearing that this might negatively affect patients’ trust. Our study, as well as previous research [34], shows that trust may actually increase when HCPs are transparent about limited or uncertain information. As patients with rare conditions often become “experts” of their own disease, they develop a high need of control over their medical condition [35], which requires transparency on the available information. This “expert role” patients with a rare condition might take on, including providing information themselves, may make physicians feel like their expertise is being questioned. However, in this study, as well as in previous research by Gómez-Zúñiga et al. [36], it was shown that trust is enhanced rather than reduced if medical specialists are receptive towards new information and seriously respond to it. Of note, our participant group included mainly highly educated patients. This could have affected how patients develop “expertise” regarding their rare cancer. Future research needs to establish to what extent patients with lower education or health literacy levels are similarly able to obtain expertise regarding their own disease and potential implications for the care they receive.

Furthermore, we identified properly coordinated care, including adequate cooperation within and between hospitals, as an important component of patients’ public and interpersonal trust. This aligns with previous work, in which “quality of cooperation” was described as a dimension of “public trust” [29]. For patients with a rare cancer, the particular importance of coordinated care may result from a lack of clear care pathways due to fragmentation of care (e.g., diagnostic and/or treatment referral to several hospitals) [7]. To ensure patients’ trust, it is important to enhance the centralization of care. In line with previous work, we found that patients experience a need for supportive care, as part of properly organized cancer care [8]. The establishment of a fixed point of contact may enhance patients’ perception of organized care and, consequently, enhance their trust in healthcare [37].

### Strengths and limitations

To our knowledge, this is the first study specifically exploring trust in-depth among patients with a rare cancer. A diverse participant group was included, consisting of patients varying in age, gender, educational level, and type of cancer. However, given the large number of rare cancer types, the participant group does not reflect all these types of rare cancer and the results need to be interpreted with caution. A limitation is that the participant group did not include patients with a non-Western migration background, for whom trust might be constructed differently. Moreover, most patients received treatment at an academic hospital or CoE, whereas patients being treated at regional hospitals may have had more negative experiences with a lack of expertise regarding their rare cancer type. This selection bias may reduce transferability of results to these groups. However, many patients in our participant group did have experiences with medical specialists and other HCPs at regional hospitals during the diagnostic process of their rare cancer and thus shared experiences regarding trust in these HCPs or hospitals as well. Finally, in the current study, experiences of patients within the Dutch healthcare system are described. Despite global challenges for patients with rare cancers, experiences may vary due to differences in healthcare systems, quality of care, travel distances, and country-specific challenges. Consequently, results should be interpreted with caution for other countries.

### Implications for practice and research

The importance of expertise for rare cancer patients’ trust and, consequently, the importance of timely referring these patients to a CoE should be acknowledged. However, it is recommended that the HCP discusses the consequences of receiving treatment in a CoE with the patient. That is, patients with a rare cancer are more likely to travel as far as necessary to receive specialized care, compared to patients with a more common cancer type. Yet, longer travel distances may also be a burden for patients and/or relatives (e.g., due to feeling sick or high costs) [7]. Further, HCPs can contribute to patients’ trust by explicitly *confirming* their expertise to patients, e.g., by communicating how often they performed a specific surgery in the last year or how many patients they treated with this specific rare cancer type. Additionally, patients should be advised to actively look up information about their HCP or hospital regarding expertise and knowledge of their specific cancer type (e.g., on the hospital’s website or through a central information point). HCPs may enhance patients’ trust through their communication, particularly by being open and transparent about the availability of information as well as uncertain aspects. However, it is important to assess individual patients’ information needs beforehand and tailor communication accordingly, since patients deal with uncertainty in different ways. Although some patients may gain hope from uncertainty, others perceive it as a threat, which may cause negative feelings [38]. Furthermore, HCPs can proactively ask patients about information they have obtained themselves and might offer to discuss the information at some point. Further development of care organization for patients with a rare cancer is needed, focusing on improving expertise as well as coordination of care within and between hospitals to build trust. Promotion of research, continuity of care, correct transfer of patient data, and adequate collaboration (of networks) are important in this regard. In addition, integrating supportive care, by establishing a fixed point of contact, can increase trust in the healthcare system. Finally, it is important that HCPs acknowledge the importance of trust and take action if they sense that their patient’s trust is not optimal. For example, they might explicitly discuss the issue and/or explore whether another HCP would be more suitable for that patient.

Future research should focus on subgroups of patients with a rare cancer. Each rare cancer type or domain may present specific challenges—for example, insufficient available patient information may be the most pressing problem for some cancer types, whereas other types may urgently need treatment guidelines. By examining trust within subgroups/domains, factors relevant to trust that specifically apply to that subgroup can be identified and tailored interventions to enhance trust can be developed.

### Conclusion

Patients with a rare cancer experience specific challenges that might influence their trust in HCPs and the healthcare system. To increase trust, it is crucial for all involved HCPs and policy makers to recognize and address these challenges by focusing on (development and confirmation of) expertise, effective provision of information, proper coordination of care, and facilitating adequate supportive care. Enhancing trust of patients with a rare cancer will benefit their experiences of care and may eventually enhance their health outcomes.

## Supplementary Information

Below is the link to the electronic supplementary material.

Supplementary file1 (PDF 1001 KB)

Supplementary file2 (DOCX 25 KB)

Supplementary file3 (DOCX 27 KB)

## Data Availability

Data are available from the corresponding author upon reasonable request.
