# Supplementary material for: Disentangling trust of patients with rare cancer in their healthcare professionals and the healthcare system: a qualitative interview study
Source: J Cancer Surviv. 2024 Jan 16;19(3):1059–68. doi: 10.1007/s11764-023-01531-w (PMC12081587; doi:10.1007/s11764-023-01531-w)
Supplement: Supplementary file 2 — Supplementary file2 (DOCX 25 KB) [file 11764_2023_1531_MOESM2_ESM.docx]

**Supplementary file**

**2. Interview guide**

| **Topics** |  |
| --- | --- |
| Introduction | Hello ..., first, I would like to thank you for participating in this study. My name is Barbara Simons and I am following the master 'Health Sciences' at the Free University of Amsterdam. This research is part of my internship at IKNL. With me is ... *introducing fellow student* |
| Aim of the study | The aim of the study is to get insight into trust of patients with rare cancer in their medical specialists, other healthcare professionals involved and the healthcare system. With the outcomes, we are looking for leads to improve trust and support for patients with rare cancers. |
| Information package | Prior to the interview, you have received an information package, containing an information letter, questionnaire and consent form for participating in the study. Do you have any questions about this? |
| Length of the interview | I would like to discuss some practical matters regarding the interview. The interview will take about 45-60 minutes. Is that feasible for you? |
| Your experience | The interview is all about your experience, so there are no wrong answers. If we move too far away from the topic of 'trust' then I may bring you back to the main topic. This is not because what you say is not interesting, but because I want to cover everything sufficiently within the time limit. |
| Anonimity and confidentiality | The interview is recorded and your data is processed anonymously. The audio recording will be erased after the study is finished. |
| Quitting | You may stop the interview/or participation in the study at any time. Also, if you prefer not to answer a question, you may indicate this. |
| Recording | Do you have any questions about what I have just told you? Then I will now start the recording. |

**Interview**

| **Trajectory**   - Diagnosis - Treatment - Challenges & positive contributions - Important healthcare professionals |
| --- |
| To start with, I would like to understand a little bit more about the trajectory you have gone through so far. This will also help me to better understand some answers later on in the interview. We will take about 10-15 minutes for that. That's not very long, but that leaves us enough time to talk about the topic of trust.   - How did your process go from first symptoms/complaints to receiving the diagnosis?   * Think of general practitioner, recognizing symptoms, referral hospital, misdiagnosis, referral other hospital, second opinion.   - Can you estimate how long it took you to get the (correct) diagnosis? (Time between first symptoms/complaints and diagnosis)   After getting (correct) diagnosis:   - Which steps did you go through to get to the (correct) treatment plan? ** Think of second opinion, referral other medical specialist/hospital, availability of treatment options (national/abroad)* - What challenges did you experience during the (diagnosis/treatment) trajectory?  ** Think of availability of information, possible search for treatment, expertise, support, cooperation (within or between hospitals)* - Are there any factors (or people) that contributed to the trajectory in a positive way? - Where do you currently stand in the trajectory? - Do you know what the next part of the trajectory will look like?   Thank you for providing insight into your trajectory. The following part of the interview will focus on the topic of trust. I would first like to ask you about trust in your medical specialist. Then we will talk about trust in other healthcare professionals involved, and finally about trust in the healthcare system. Several medical specialists or healthcare professionals may be or have been involved in your trajectory. Therefore, I first want to agree with you on who we will discuss.   - Which medical specialist(s) is/are the key person(s) in your trajectory? - Who was/is the most important? (function, not the name) - What was his/her role? - What other healthcare professionals were/are involved in your trajectory? (E.g. general practitioner, nurse, case manager) - Which healthcare professionals were or are of most importance to you within this trajectory?  *In case of many healthcare professionals, ask for two of the most important, besides the most directly involved medical specialist |

| **Trust in general** |
| --- |
| I would now like to address the topic ‘trust’ within the trajectory.   - What does trust mean to you, in general? - What does trust mean to you as a person (by this I mean e.g. are you someone who quickly trusts someone (something) or not?) - Can you explain what caused this way of trusting / how it formed?   **Think about personality, coping, caution towards others, past experiences (in general, but also in relation to being ill)* |

| **Trust in medisch specialist**   - Level of trust including barriers and facilitators |
| --- |
| We just discussed that (medical specialist) was/is a key person in your trajectory. *If there are several, agree who will be discussed first.   - To what extend do you trust your/this medical specialist? - How did trust in this medical specialist develop throughout the trajectory?  - Was trust there from the beginning?  - Has trust in your medical specialist changed?  - How did that happen? - Were there specific things that were hindering or boosting trust in your medical specialist?  ** Think of information provision/expertise/* - *support/cooperation with other healthcare professionals* - How did that affect trust? (became more/less?) - How did you cope with that?/how do you cope with that?  *If participant has very high or very low trust:* - What might reduce trust in your medical specialist? - What do you need to increase trust in your medical specialist?   *If there was another medical specialist who was a key person*  You have just mentioned that in addition to *medical specialist 1*, *medical specialist 2* was or is a key person in your trajectory.   - Compared to medical specialist 1, is the level of trust different for medical specialist 2? - What is different? - Why is that? - Did trust in medical specialist 2 develop in a different way during the trajectory, compared to medical specialist 1? - With medical specialist 2, were there other things that were hindering or boosting trust?  ** Think of information provision/expertise/support/cooperation with other healthcare professionals* - In what way was it different? How did that affect trust? - How did you cope with that?/how do you cope with that? |

| **Level of trust in other healthcare professionals involved**   - Level of trust including barriers and facilitators - Importance and consequences of trust |
| --- |
| At the beginning of the interview, you mentioned a number of other involved healthcare professionals who are or were important to you. I would now like to discuss trust in these healthcare professionals. Shall we start with *healthcare professional*?   - What was their role? - Compared to *medical specialist(s)*, is the level of trust different for *healthcare professional*? - What is different? - Why is that? - Did trust in *healthcare professional* develop in a different way during the trajectory, compared to *medical specialist(s)*? - With medical specialist 2, were there other things that were hindering or boosting trust?  ** Think of information provision/expertise/support/cooperation with other healthcare professionals* - In what way was it different? How did that affect trust? - How did you cope with that?/how do you cope with that?   If another important healthcare professional was involved:  Another healthcare professional who is/was important during your trajectory was *healthcare professional*.   - What was their role? - Compared to *medical specialist(s) and other healthcare professionals*, is the level of trust different for *healthcare professional*? - What is different? - Why is that? - Did trust in *healthcare professional* develop in a different way during the trajectory, compared to *medical specialist(s) and other healthcare professionals*? - With *healthcare professionals*, were there other things that were hindering or boosting trust?  ** Think of information provision/expertise/support/cooperation with other healthcare professionals* - In what way was it different? How did that affect trust? - How did you cope with that?/how do you cope with that? - How important is trust in your medical specialist and other healthcare professionals to you? - For what reason is trust important? - Do you experience consequences of having high/low trust in your medical specialist(s) and other healthcare professionals? If yes, in what ways? *** *Think of communication, decision-making, feelings, satisfaction, adherence to advice* - Can you explain how trust affects this? - Does it matter that the level of trust may differ between healthcare professionals? |

| **Dimensions of trust** |
| --- |
| It is known from previous research that there are a number of characteristics that can be important for a person's trust in the physician. I have already heard some of these characteristics in this interview, such as *depending on what participant said*. Other characteristics are *name only those that have not yet been mentioned*:  - Competence (the medical skill of the physician)  - Loyalty (that the physician puts your interest as a patient first)  - Honesty (Honesty and openness of the physician)  - Caring (the physician's involvement and compassion)   - How important is *dimension* for trust in the medical specialist, for you? - Can you explain why you think it is or is not important for trust in the medical specialist? - If you think of *other healthcare professional*, are the same characteristics important? - Can you explain this? - Are any other characteristics important for trust in your medical specialist or healthcare professionals? If yes, which ones? |

| **Trust in the healthcare system**   - Level of trust - Importance/consequences of trust - Relationship between trust healthcare system and medical specialists/healthcare professionals |
| --- |
| We have just discussed trust in the medical specialist and other healthcare professionals. Besides trust in your healthcare professionals, I am also very interested in your trust in the healthcare system. By this we mean the organization of care for patients with a rare cancer.   - To what extend do you trust the healthcare system? - Are there specific things that have affected your trust in the healthcare system?  ** Think information provision/insurance issues/access to treatments and expertise/availability of support/collaboration between professionals or hospitals/ whether or not you have a fixed point of contact/patient organizations* - o How has this affected your trust? (More/less) - o Has your trust in the healthcare system changed during the process? Why is that? - o How do you deal with that? /How have you dealt with that? - o In case of low trust: What would you need to increase trust in the care system? - Does having high/low of trust in the healthcare system affect trust in the medical specialist and other healthcare professionals? In what ways? - Does low/high trust in the medical specialist and other healthcare professionals affect trust in the healthcare system? In what ways? |

**Closing**

| Closing | Then I would like to finish the interview in a moment, I have already received a lot of useful information. Are there any topics that have not yet been discussed but that you think are relevant to mention? |
| --- | --- |
| Summary | Then I would like to briefly summarise what we discussed:  *Provide brief summary*  Is that correct? Do you have anything else to add? |
| Thanks | Then I would like to say thank you for your time and participation in the study. If you have any questions or comments later on about the interview or participation in the study, please feel free to contact me again. I would also like to wish you the best of luck with your situation. |
