# Supplementary material for: Disentangling trust of patients with rare cancer in their healthcare professionals and the healthcare system: a qualitative interview study
Source: J Cancer Surviv. 2024 Jan 16;19(3):1059–68. doi: 10.1007/s11764-023-01531-w (PMC12081587; doi:10.1007/s11764-023-01531-w)
Supplement: Supplementary file 3 — Supplementary file3 (DOCX 27 KB) [file 11764_2023_1531_MOESM3_ESM.docx]

**Supplementary file**

**3. Overview codes**

| Trust | | | |
| --- | --- | --- | --- |
| Expertise | Information | Continuity of care | Support |
| Meaning:   - Competence   Trajectory:   - Expert care   Dimension:   - Competence - Competence/   expertise difficult to assess   - Honesty   Facilitator:   - Transparancy - Knowledge - Experience - Seeing result of treatment - Being part   of experimental treatment   - Expertise - Communication- techniques - Cooperation - Trust that hospital is doing what they can - Based on   what the doctor has achieved   - Confidence - Expertise-   (centres)  Barrier: Not thinking out of the boxNot taking complaints seriouslyLittle knowledgeNo cooperationDoctor in training | Importance:Patient's life depends on itPatient knows too little about it Meaning:   - Honesty - Confidentiality   Trajectory:   - Not recognising symptoms cannot be blamed - Much is unknown - Delayed diagnosis   Facilitator:   - Confident - Openness - Collaborative attitude - Honesty - Correct info - Foreign studies - Expertise - Direct - Clarity - Being open for information - Convincing - Responding to questions - Calm - Transparency - Protocols - Cooperation - Communication techniques   Barrier:   - Lack of clarity - Advice not based on guideline - Withholding info - Not open towards info - Incorrect information - Little money - Little research - Little information | Meaning:   - Wishing the best for the patient - Taking it seriously - Feeling heard   Trajectory:   - Expert care - Varying   doctors  Facilitator:   - Taking action if required - More frequent contact - Seeing the same doctors - Taking complaints seriously - Organization in the hospital   Barrier:   - Varying doctors - Little knowledge - Not responding to request - No cooperation - Unclear transfer of information - Not accessible - Complaints not recognised in a timely manner | Meaning:   - Being able to say anything to anyone - Empathy - Feeling - Listening - Being able to count on someone - Support after diagnosis   Trajectory:   - Managing a lot yourself - Assertiveness - Lack of support   Dimension:   - Loyalty - Caring   Facilitator:   - Stick to agreement - Support - Physical appointments - More frequent contact - Quality of life comes first - Commitment - Taking time - Empathy - Not a number - Interest - Equality - Accessibility - Taking action if necessary - Commitment - Feeling - Shared decision-making - Thinking together - Being involved - Take it seriously   Barrier:   - Lack of support - Less interest - Not taking time - Lack of time - Not empathetic - Not actively suggesting options - Pt needs to be empowered - businesslike |
| Other codes | | | |
| Trajectory in general:   - Patients' organization - Travel time - Went fast - No choices - Second opinion - Careful assessment - Talking is important   Attitude towards trust:   - Just until it is breached - Intuitive - Not trustworthy - Neutral - Must be earned   Consequences low/high trust:   - Being in control - Following advice - Checking more - Rest - Do not question - Switching - Don’t feel like going - Getting through the process easily - Being able to tell everything   Facilitating trust:   - Correct handling of data - Healthcare system   Barrier for trust:   - Organization development hospital | | | |
